# Determination of Bioactive Compounds in Sequential Extracts of Chia Leaf (*Salvia hispanica* L.) Using UHPLC-HRMS (Q-Orbitrap) and a Global Evaluation of Antioxidant In Vitro Capacity

**DOI:** 10.3390/antiox10071151

**Published:** 2021-07-20

**Authors:** María Carolina Zúñiga-López, Gabriela Maturana, Guillem Campmajó, Javier Saurina, Oscar Núñez

**Affiliations:** 1 Department of Inorganic and Analytical Chemistry, Faculty of Chemical and Pharmaceutical Sciences, University of Chile, Sergio Livingstone, 1007, Independencia, Santiago 8380492, Chile; gamaturana@uchile.cl; 2 Department of Chemical Engineering and Analytical Chemistry, University of Barcelona, Martí i Franquès 1-11, E08028 Barcelona, Spain; gcampmajo@ub.edu (G.C.); xavi.saurina@ub.edu (J.S.); 3 Research Institute in Food Nutrition and Food Safety, University of Barcelona, Av. Prat de la Riba 171, Edifici de Recerca (Gaudí), E08921 Santa Coloma de Gramenet, Spain

**Keywords:** *Salvia hispanica* L., antioxidant capacity, leaves extracts, ORAC, Folin–Ciocalteu, DPPH, UHPLC-HRMS

## Abstract

Consumers’ interest in foods that are nutritionally balanced and with health benefits has increased. The food industry is paying attention to the use of the ancestral seed *Salvia hispanica* L., commonly known as chia. At present, only chia seeds, which are a natural source of omega-3 and omega-6, fiber, proteins, and natural antioxidants, are commercialized. Although some studies reveal the presence of several bioactive compounds, such as polyphenols (e.g., vitexin, orientin, and some hydroxycinnamic acids) in chia leaf methanolic extracts, the chia plant is commonly used as fertilizer or treated as waste after harvest. Therefore, it can represent a by-product that could be considered a great source of bioactive compounds with unexplored potential in medicine and food industry applications. In this work, UHPLC-HRMS (Q-Orbitrap) was employed to tentatively identify and determine the bioactive compounds present in different leaf extracts of chia plants of black and white seed phenotype obtained with solvents of different polarity (ethanol, ethyl acetate, dichloromethane, and hexane) to address chia plant by-product revalorization. The chemical antioxidant capacity was also studied and correlated to the found bioactive compounds. In these experiments, black chia showed a higher antioxidant capacity than white chia in the ethanolic extracts. Moreover, experiments on cellular antioxidant activity were also performed with a predominance of the white chia extract. It is noted that the cellular antioxidant activity results make chia ethanolic extracts promising antioxidants.

## 1. Introduction

*Salvia hispanica* L., commonly known as chia, is an herbaceous plant belonging to the Lamiaceae family, native to southern Mexico [1]. The ancestral seeds of chia are widely known in the food industry due to their high nutritional value. Particularly, they possess high levels of omega–3 fatty acids in α-linolenic acid, which constitute between 24% and 30% of the seed’s weight [2,3,4,5,6,7], and secondary metabolites to a lesser extent [8,9]. In this context, some of the secondary metabolites found in chia seed are polyphenols—such as gallic acid, caffeic acid, quercetin, kaempferol, and catechin—with concentrations of 0.5 and 0.8 mg of total polyphenols per gram of seed in the crude and hydrolyzed seed extracts, respectively [5,8,9,10].

In recent years, polyphenols have gained more attention due to their nutraceutical uses in cardiovascular diseases, diabetes, and cancer, among others [11]. Moreover, polyphenols also have antiviral, antifungal, anticancerogenic, antitumoral, and anti-inflammatory properties [11,12].

Considering the consumer’s interest in healthy and nutritional foods, the cultivation of this crop has been globally extended, focusing on chia seed production. In this line, alongside its increased seed production, research in chia seed is vast and diverse, centering on its properties and characteristics [7,13,14]. On the contrary, chia plant roots and aerial parts have been overridden and treated as waste or used as soil fertilizer, despite the folkloric knowledge given by the ancient civilizations of the Mesoamerican zone. For instance, although no scientific evidence has ever been provided, roots were formerly used to treat diarrhea, while leaves were used to treat skin lacerations [1,15]. 

Unlike chia seed research, a few studies deal with chia leaves composition and antimicrobial properties [8,16]. On the one hand, Amato et al. reported the tentative identification of 34 polyphenols—including, among others, rosmarinic acid, caffeic acid, orientin, vitexin, apigenin, and quercetin—by high-performance liquid chromatography coupled to mass spectrometry in methanolic extracts obtained by the maceration of the vegetal matrix [8]. On the other hand, Elshafie et al. [16] reported that the principal constituents of essential oil extracted from aerial parts of this plant were sesquiterpenes with a majority of caryophyllenes. Moreover, the authors concluded that this product could be potentially used for microbial control regarding the antimicrobial effect.

The present study aimed to explore the antioxidant capacity (AC) of chia leaves by three different methods to consider the variability of AC quantification in natural products. Thus, Oxygen Radical Absorbance Capacity (ORAC), 2,2′-diphenyl-1-picrylhydrazyl (DPPH), and a modified ORAC procedure were applied. ORAC and DPPH are widely used methodologies, whereas the third method appears promising to capture the reactivity of the antioxidants [17]. Additionally, assays on cellular antioxidant activity (CAA) were performed on the extracts with the best results in chemical AC analyses described. 

Moreover, this study also aimed to identify the phenolic composition by ultra-high-performance liquid chromatography coupled to high-resolution mass spectrometry (UHPLC-HRMS) (Q-Orbitrap) and evaluate the antioxidant capacity (ORAC, DPPH) and total phenolic content (TPC), through the Folin–Ciocalteu (FC) method, of two seed phenotypes leaves extracts, obtained using different polarity solvents.

## 2. Materials and Methods

### 2.1. Cultivation, Sampling and Drying of Chia Plants

The plants used in this study were cultured at the experimental field INIA Intihuasi, belonging to the University of Chile, near La Serena city (Chile) (28°34′41.01′′ S–70°47′52.62′′ O). The culture was done between January and June 2015 by triplicate for each phenotype (black and white seed). The samples were taken to the laboratory within the same day, and the leaves were separated, labeled, and dried at 35 °C in the stove until constant weight. After the drying process, the leaves were crushed with a mortar and stored at room temperature in darkness.

### 2.2. Extraction

In order to obtain an exhaustive characterization of the black (B) and white (W) seed phenotypes chia leaves samples and broaden the molecular coverage, sample extracts were obtained after successive extractions with different organic solvents: hexane (HEX), dichloromethane (DCM), ethyl acetate (EA), and ethanol (EtOH). Thus, eight different sample extracts, comprising B-HEX, B-DCM, B-EA, and B-EtOH for chia leaves of black seed phenotype, and W-HEX, W-DCM, W-EA, and W-EtOH for chia leaves of white seed phenotype, were analyzed.

Each extraction was carried out until the matrix was completely exhausted. Between each extraction, the matrix was dried at room temperature before adding the new dissolvent. The sequential extracts were concentrated in a rotary evaporator at reduced pressure. Once the extract was dried, it was dissolved in methanol to 5000 mg dried extract per liter.

Moreover, global extractions were done to compare AC with other natural products. For this purpose, extractions of the chia plant leaves of black and white seed phenotypes were performed using the acetone:water:acetic acid (AWA) (70:29.5:0.5 *v*/*v*/*v*) mix, following the procedure described by Wu et al. [18].

### 2.3. Chemicals and Reagents

AAPH (2,2-azobis (2-methylpropionamidine) dihydrochloride), fluorescein (as disodium salt) (3′,6′-dihydroxyspiro[isobenzofuran-1(3H),9′-[9H]xanthen]-3-one), pyrogallol red (3′,4′,5′,6′-Tetrahydroxyspiro[benzo[c][1,2]oxathiole-3,9′-xanthene]1,1-dioxide), Trolox (6-hydroxy-2,5,7,8-tetramethylchroman-2-carboxylic acid), 2′,7′-dichlorodihydrofluorescein diacetate (DCFH_2_-DA), gallic acid (3,4,5-trihydroxybenzoic acid), rosmarinic acid 3-(3,4-dihydroxyphenyl)-2-{[(2E)-3-(3,4-dihydroxyphenyl)prop-2-enoyl]oxy}propanoic acid, caffeic acid (CA) ((2E)-3-(3,4-Dihydroxyphenyl)prop-2-enoic acid), DPPH (1,1-Diphenyl-2-picrylhydrazine), Folin–Ciocalteu (FC) reagent, and sodium carbonate were purchased from Sigma-Aldrich (St. Louis, MO, USA). Milli-Q water and phosphate buffer were used for dilutions for all measurements.

LC-MS grade water, acetonitrile, and formic acid (98–100%) were purchased from Sigma-Aldrich (Steinhein, Germany).

### 2.4. Antioxidant Capacity

For the chemical AC and FC assays, a 5000 mg L^−1^ solution of the dry extract was prepared in methanol for each of the eight chia extracts. 

#### 2.4.1. ORAC-FL Assay

ORAC-Fluoresceine (ORAC-FL) assay was evaluated following the method described by Ou et al. [19]. Briefly, 2 µL of each chia methanolic extract solution were diluted in 998 µL of 75 mM sodium phosphate buffer (pH 7.4). Then, 25 µL of each diluted extract was mixed with 150 µL of 40 nM of fluorescein in phosphate buffer in a 96-well white polystyrene microplate. Blanks were prepared as samples but with 25 µL phosphate buffer instead of the sample. The microplate was placed in a Synergy HT multi detection microplate reader (Bio-Tek Instruments, Winooski, Vermont) and then incubated for 7 min at 37 °C. The radical reaction was initiated upon the addition of 25 µL of an 18 mM solution of AAPH in phosphate buffer, using a multichannel pipette. Then, fluorescence on each well was recorded every 1 min for 240 min, after gentle shaking, by measuring the emissions from the top of the microplate, at an excitation wavelength of 485 nm and an emission wavelength of 520 nm. The time series of fluorescence decay was integrated, and the resulting area under the curve (AUC) was normalized by subtracting the AUC obtained in the blank experiment. After plotting, the sample AUC was compared with that obtained for a Trolox, and the results were expressed as an ORAC index calculated with Equation (1).

(1) $$\mathrm{ORAC}-\mathrm{FL}\;\mathrm{index}=\frac{{(\mathrm{AUC}}_{\mathrm{Sample}}{-\mathrm{AUC}}_{\mathrm{Control}})}{{(\mathrm{AUC}}_{\mathrm{Trolox}}{-\mathrm{AUC}}_{\mathrm{Control}})}\times \frac{\left[\mathrm{Trolox}\right]}{\left[\mathrm{Sample}\right]}$$

For global extractions, results were expressed as µmol Trolox Equivalent (TE) per 100 g dw (µmol TE/100 g dw).

#### 2.4.2. ORAC-PGR Assay

ORAC-Pyrogallol red (ORAC-PGR) assay was measured following a similar protocol to the one described above for ORAC-FL, based on the method of Alarcón et al. [17]. Ten µL of each chia methanolic extract solution were diluted in 990 µL of phosphate buffer, then 25 µL of diluted extracts were mixed with 150 µL of a 70 µM solution of pyrogallol red, dissolved in buffer phosphate. After 7 min of incubation at 37 °C, 25 µL of AAPH dissolved in buffer to a concentration of 0.1 M was added to the wells to initiate the reaction. The absorbance at 540 nm was measured every 35 s for 90 min from the bottom of the wells. The resulting absorbance profiles were processed as described for ORAC-FL, and the ORAC-PGR index was calculated with Equation (1). 

For global extractions, results were expressed as µmol Trolox Equivalent (TE) per 100 g dw (µmol TE/100 g dw).

#### 2.4.3. DPPH Scavenging Capacity Assay

DPPH scavenging capacity was spectrophotometrically monitored as described elsewhere [20]. In total, 20 µL of each chia methanolic extract solution were added to 2880 µL of a DPPH solution, which was prepared daily in a concentration of 50 µM or the one necessary to reach an absorbance of 0.9 units at 517 nm. After 20 min of reaction, the time in which the reaction stabilizes, changes in absorbance at 517 nm were registered for each extract. The results were expressed as inhibition percentage (Equation (2)).

(2) $$\%\;\mathrm{Inhibition}=\left(\frac{{\mathrm{Abs}}_{\mathrm{Control}}{-\mathrm{Abs}}_{\mathrm{Sample}}}{{\mathrm{Abs}}_{\mathrm{Control}}}\right)\times 100$$

#### 2.4.4. Cellular Antioxidant Activity (CAA)

Cellular antioxidant activity (CAA) was evaluated in VERO cells using 2′,7′-dichlorodihydrofluorescein diacetate (DCFH_2_-DA) as fluorescent probe. The cells were plated in white sterile polystyrene flat-bottom 96-well microplates (Nunc, Denmark) at a concentration of 50,000 cells per well and incubated for 24 h at 37 °C and 5% CO_2_ in RPMI 1640 culture medium. The cells were washed with 150 mL of PBS, pH = 7.4 and incubated for 1 h with 100 µL of RPMI 1640 containing 25 mM of DCFH_2_-DA. The extracts were added at final concentrations of 10 ppm. After 1 h incubation, the medium was discarded, and the cells were gently washed twice with 200 µL of PBS. Then, they were incubated with AAPH at a final concentration of 600 mM in phosphate-buffered saline (PBS). Fluorescence was measured immediately after AAPH addition in an EnSpire from PerkinElmer in 96-well plates at 37 °C using an excitation of 485 nm and an emission of 538 nm. The evaluation was carried out every min for 1 h and the CAA values were calculated by Equation (3) [21]:

(3) $$\mathrm{CAA}\%=\left(\frac{\Delta F-\Delta F_{\mathrm{AH}}}{\Delta F}\right)\times 100$$

ΔF = fluorescence intensity in the presence of free radical, without extracts. ΔF_AH_ = fluorescence intensity in the presence of free radical and the extracts. All measures were performed during the same period.

### 2.5. Phenolic Composition

#### 2.5.1. Total Phenolic Content

The TPC was estimated with the FC method [22]. The reaction between the extracted polyphenols and the FC reagent was carried out in a microplate reader (EnSpire, multilabel reader Perkin Elmer, Singapore, Singapore) with a 96-well polystyrene transparent multi-plate. First, samples were prepared by dissolving 40 µL of extract in 1 mL nano pure water. Then, 200 µL of the FC reagent were added in each well with 40 µL of 10% Na_2_CO_3_ solution, 45 µL of nano pure water, and 15 µL of the corresponding sample (final total volume of 300 µL). For blank samples, 15 µL nano pure water was added instead of the sample extract. Finally, the absorbance at 765 nm was measured after 30 min at 37 °C. The assay was performed in triplicate for each extract. The results, obtained using linear interpolation between the net absorbance and the calibration curve of gallic acid in the range of concentrations 0.98–12.04 mg L^−1^, were presented as milligrams per liter gallic acid equivalents (mg GAE L^−1^). They were finally expressed in mg GAE/100 g dry weight (dw) chia leaves.

#### 2.5.2. Caffeic and Rosmarinic Acid Analysis

Caffeic and rosmarinic acid quantification was performed on an Agilent Technologies 1200 HPLC system (Waldbronn, Germany) with a C18e (100 × 4.6 mm I.D.,) Chromolith^®^ HighResolution column (Merck, Darmstadt, Germany) and a Diode Array Detector (DAD). The mobile phase flow rate was set at 1 mL min^−1^ and two mobile phase components were used (A: 2% acetic acid aqueous solution; B: acetonitrile) with the following mobile phase gradient program: 0–2 min, 92–91% A; 2–4 min, 91–89.8% A; 4–6 min, 89.8–88.8% A; 6–8 min, 88.8–87.5% A; 8–10 min, 87.5–86.5% A; 10–12 min, 86.5–85.2% A; 12–14 min, 85.2–84.1% A; 14–16 min, 84.1–83.0% A; 16–18 min, 83.0–81.2% A; 18–20 min, 81.2–80.9% A; 20–23 min, 80.9–75.0% A; 23–25 min, 75.0–70.0% A; 25–28 min, 70.0–65.0% A; 28–30 min, 65.0–62.0% A. 

A 5000 mg L^−1^ chia extract methanolic solution was injected with an injection volume of 20 µL. The chromatograms were registered at 278 nm and 322 nm. The identification was performed by comparison of the retention time and the absorbance spectra with the respective standard. Quantification was done by means of a caffeic acid (CA) calibration curve (0.1–1 mgL^−1^; LOD 0.02 mgL^−1^; LOQ 0.07 mgL^−1^) and rosmarinic acid (RA) calibration curve (0.5–10 mgL^−1^; LOD 0.19 mgL^−1^; LOQ 0.65 mgL^−1^).

#### 2.5.3. UHPLC-HRMS (Q-Orbitrap) Phenolic Identification

Chromatographic separation for the phenolic identification on the chia methanolic extracts under study was performed on an Accela UHPLC system (Thermo Fisher Scientific, San Jose, CA, USA) equipped with a quaternary pump, an autosampler, and a column oven. A porous-shell Ascentis Express C18 reversed-phase column (150 × 2.1 mm, 2.7 µm partially porous particle size) from Supelco (Bellefonte, PA, USA) was used under gradient elution based on (A) water and (B) acetonitrile (both containing 0.1% formic acid) as the mobile phase components, and following the next elution program: 0–1 min, isocratic elution at 10% solvent B; 1–20 min, linear gradient from 10% to 95% solvent B, 20–23 min isocratic elution at 95% solvent B; 23–24 min, back to initial conditions; from 24 to 30 min, column re-equilibration at 10% solvent B. The mobile phase flow rate was set at 300 µL min^−1^ and an injection volume of 1 µL (in full loop mode) was employed.

The UHPLC system was coupled to a Q-Exactive Orbitrap HRMS instrument (Thermo Fisher Scientific) with a heated electrospray ionization source (HESI-II), operating in negative ionization mode with a capillary voltage of −2.5 kV. Nitrogen was employed for the sheath, sweep and auxiliary gases at flow rates of 60, 0, and 10 au (arbitrary units), respectively. HESI-II heater temperature was set at 350 °C. Instrument capillary temperature was set at 320 °C, and an S-Lens RF level of 50 V was employed. Q-Exactive HRMS Orbitrap was tuned and calibrated using commercially available Thermo Fisher calibration solutions every 3 days. Full MS scan mode with a *m*/*z* range from 100 to 1500 at a mass resolution of 70,000 full width at half-maximum (FWHM, at *m*/*z* 200), with an automatic gain control (AGC) target of 1.0 × 10^6^ and a maximum injection time (IT) of 200 ms, was employed. For fragmentation, a data-dependent acquisition (DDA) mode was used, operated in product ion scan mode and applying stepped normalized collision energies (NCE) of 17.5, 35, and 52.5 eV. The product ion spectra were registered with an isolation window of 0.5 *m*/*z* and a fixed first mass of *m*/*z* 50. In this case, a mass resolution of 17,500 FWHM (at *m*/*z* 200), with an AGC target of 2.5 × 10^5^, and a maximum IT of 200 ms, was used. DDA mode was activated when a signal higher than an intensity threshold of 1.0 × 10^5^ was detected in the full MS scan mode. Control of the UHPLC-ESI-HRMS system and data processing were carried out by using Xcalibur^TM^ 2.2 software (Thermo Fisher Scientific).

Phenolic screening and identification in the analyzed chia methanolic extracts were carried out by processing the UHPLC-HRMS raw chromatographic data with TraceFinder^TM^ version 3.3 software from Thermo Fisher Scientific and using a homemade accurate mass database list (Table S1) attached in the Supplementary Materials. In all cases, confirmation criteria to assess the tentative presence and identification of a targeted phenolic compound in the analyzed samples relied on accurate mass errors (values below 5 ppm) and isotopic pattern matches (scores higher than 85%). Besides, an additional confirmation criterion based on the chromatographic retention time was also employed for those phenolic compounds for which the commercial standard was available in the laboratory.

### 2.6. Statistical Analysis

Evaluation of significant differences between extracts for each analysis of AC and phenolic composition was made with a one-way ANOVA, and the Statgraphics centurion software was used to calculate the *p* value and *p* < 0.05 was considered as significant difference.

## 3. Results

### 3.1. Extraction

Sequential extractions were performed with 13.7149 g of dry leaf mass for black seed phenotype and 6.6056 g of dry leaf mass for white seed phenotype. Table 1 summarizes the extraction ratio for each extract obtained by comparison of the initial leaf dry mass and the dry extract mass.

The total extraction ratio was 51.185% for leaves of black seed phenotype and 68.172% for leaves of white seed phenotype. For both seed phenotypes, the highest extraction ratio was achieved with hexane (B-HEX and W-HEX) followed by the ethanolic extracts (B-EtOH and W-EtOH).

### 3.2. Leaf Antioxidant Capacity

In ORAC measures, the AC is evaluated through a reaction between the antioxidants, a probe, and the oxygen-centered radicals generated by the thermal decomposition of AAPH. The oxygen-centered radicals react with a probe (fluorescein/pyrogallol red), leading to an oxidized product with different spectral behavior, allowing fluorescence/absorbance detection [17,19]. ORAC-FL and ORAC-PGR methodologies have the same mechanism (hydrogen atom transfer), but the outcome depends on the molecular probe. This allows complementation of both responses considering not only the stoichiometry (ORAC-FL) but the reactivity (ORAC-PGR) of antioxidants present in the different extracts [17].

**Figure 1:**
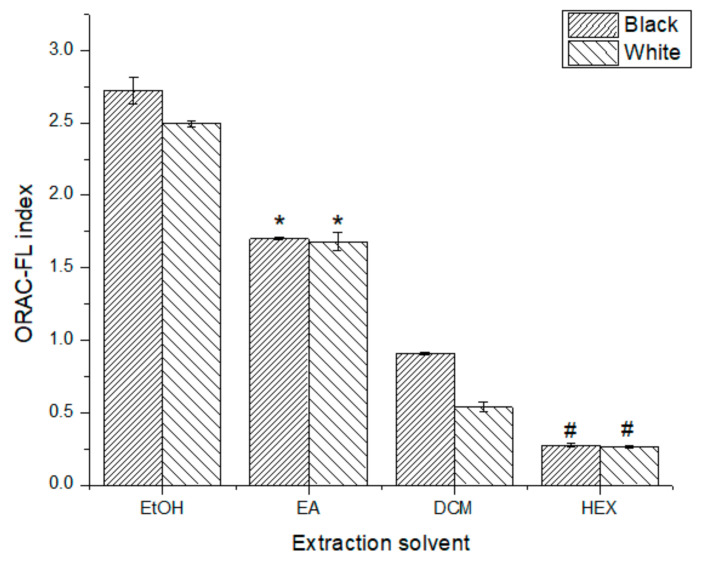
ORAC-FL index for all extracts tested. *, # No significant difference (*p* > 0.05).

According to the ORAC-FL index values summarized in Figure 1, the extracts carried out with more polar solvents (EtOH and EA) presented a higher AC than the extracts done with less polar solvents (DCM and HEX), with the B-EtOH extract having the highest ORAC-FL index (2.72 ± 0.09). In fact, the decrease in AC with the decrease in the polarity of the solvent was statically significant (*p* < 0.05) for both phenotypes. For the EtOH and DCM solvents, the black seed phenotype leaf extracts (B-SPLE) had a significantly higher AC (*p* < 0.05) than the respective white seed phenotype leaf extract (W-SPLE). The EA and HEX extracts were the only ones with no significant differences between phenotypes (*p* > 0.05).

**Figure 2:**
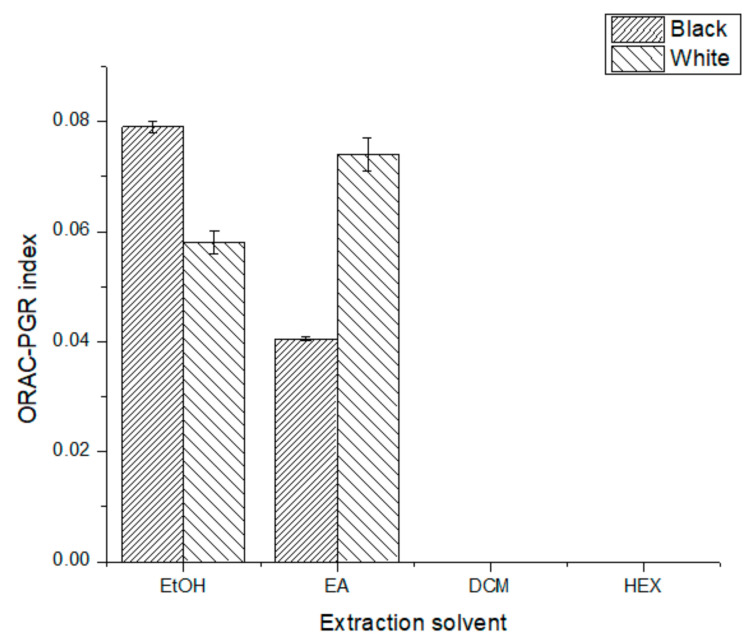
ORAC-PGR index for all extracts tested. All differences were statically significant (*p* < 0.05).

In order to compare with available literature data, ASE global extractions were performed following the protocol reported by Wu et al. [18], using AWA as an extractant. An average ORAC-FL AC of 24,061 ± 2609 µmol TE/100 g of dw was found for B-SPLE and 9973 ± 3128 µmol TE/100 g of dw for W-SPLE. For the ORAC-PGR AC assay (Figure 2), the concentration of HEX and DCM extracts was doubled (10,000 mg L^−1^) for both phenotypes; even so, these extracts did not mark antioxidant reactivity. Again, the best extract was B-EtOH (0.079 ± 0.001 followed by W-EA (0.074 ± 0.003). For global extraction (AWA), a mean of 371 ± 99 µmol TE/100 g of dw for B-SPLE and 381 ± 22 µmol TE/100 g of dw for W-SPLE were calculated.

ORAC assays are described as a Hydrogen Atom Transfer methodology, while DPPH inhibition in a methanolic medium is that of an Electron Transfer [23]. DPPH is a relatively stable radical with an absorbance maximum at 517 nm. This band disappears in the reduction reaction product enabling the spectrophotometric AC determination [23]. 

**Figure 3:**
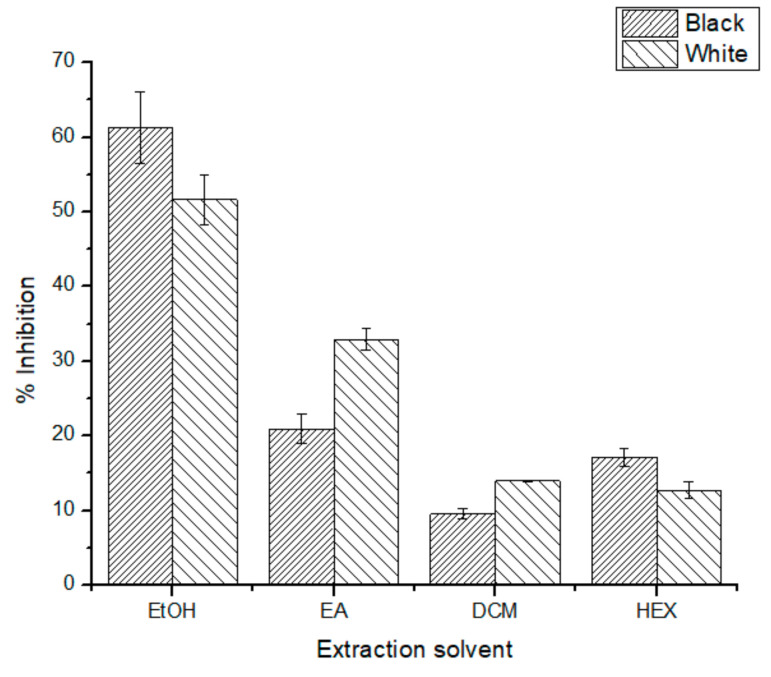
%Inhibition for all extracts tested. All differences were statically significant (*p* < 0.05).

Again, the AC measurement for all extracts was possible (Figure 3), the ethanolic extracts being those with the highest %Inhibition for both seed phenotypes with B-EtOH, having a %Inhibition of 61 ± 5% and W-EtOH of 52 ± 3%. DPPH data indicate a significant difference between all samples (*p* < 0.05) and, as well as ORAC results, the ethanolic extracts were those with the highest AC for both phenotypes.

Considering all AC data, the leaf ethanolic extract of black seed phenotype presents the best antioxidant quality, considering the stoichiometry and reactivity of the antioxidants present in the extracts.

Additionally, for the global extraction performed with AWA, average % Inhibition values of 28.2 ± 0.8 and 18.7 ± 0.6 were found for B-SPLE and W-SPLE, respectively.

**Figure 4:**
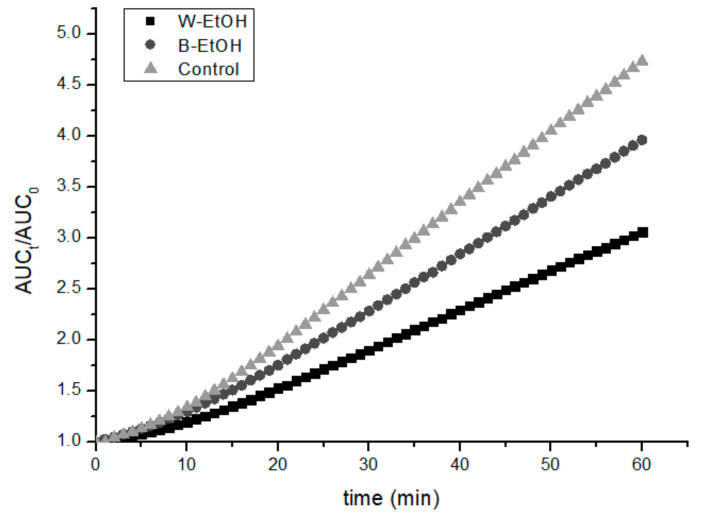
Cellular antioxidant activity of ethanolic extracts in VERO cells.

Since the ethanolic extracts were those with the highest chemical AC, an assay on cellular antioxidant activity was performed for these samples. This assay evaluates the overall oxidative profile of the cell by using DCFH_2_-DA as a probe for its detection. This molecule diffuses passively into the cell, where it is transformed into DCFH_2_ (non-fluorescent) by intracellular esterase activity, then it is oxidized to DCF by intracellular reactive oxygen species. This final product is fluorescent (λ _excitation_ = 498 nm; λ _emission_ = 522 nm), and, as the fluorescence increases, so does the oxidation of the probe. Additionally, AAPH was employed as a system stressor and VERO cells were used to assess the CAA. Figure 4 shows the increase in fluorescence for the control and both extracts tested. For this experiment, the %CAA for W-SPLE was 28 ± 2 and 13.2 ± 0.6 for B-SPLE.

### 3.3. Phenolic Composition

**Figure 5:**
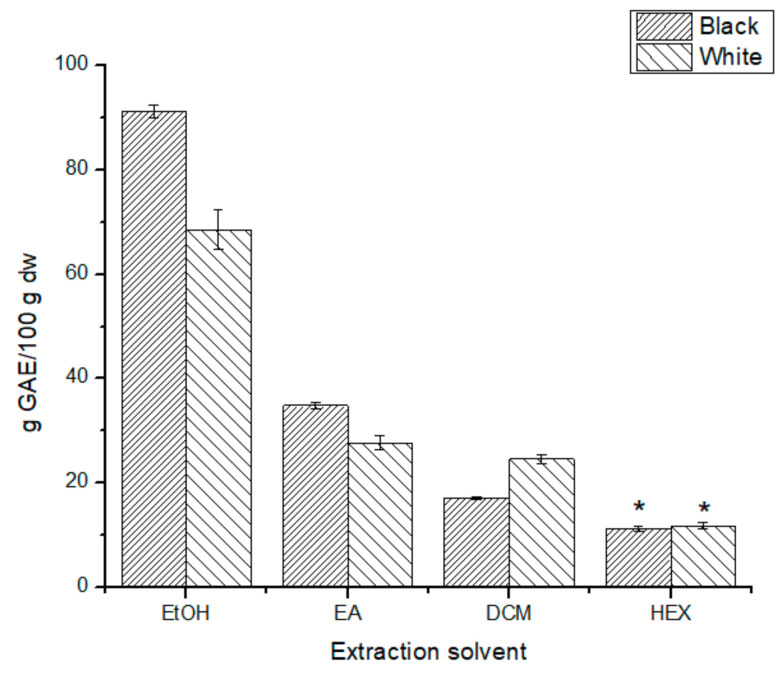
Total phenolic content for all extracts tested. * No significant difference (*p* > 0.05).

The TPC assay is based on the Folin–Ciocalteu reduction by polyphenols, which implies a color change from yellow to the blue of the FC reagent. Hence, the highest is the absorbance at 745 nm, the highest the polyphenolic content. This assay can overestimate the concentration of polyphenolic compounds if there are other reductant species [24]. According to the data shown in Figure 5, the ethanolic extracts had the highest TPC for both phenotypes (91 ± 1 g GAE/100 g dw and 68 ± 4 g GAE/100 g dw for B-SPLE and W-SPLE, respectively), and again, the TPC was significantly higher (*p* < 0.05) for the B-SPLE than W-SPLE except for the HEX extracts. The TPC significantly decreased as the polarity of the solvent decreased.

As for the global extractions, the TPC for B-SPLE was 2.0 ± 0.1 mg GAE/100 g dw and 1.71 ± 0.06 mg GAE/100 g dw for the W-SPLE, much lower than the ethanolic extracts.

**Figure 6:**
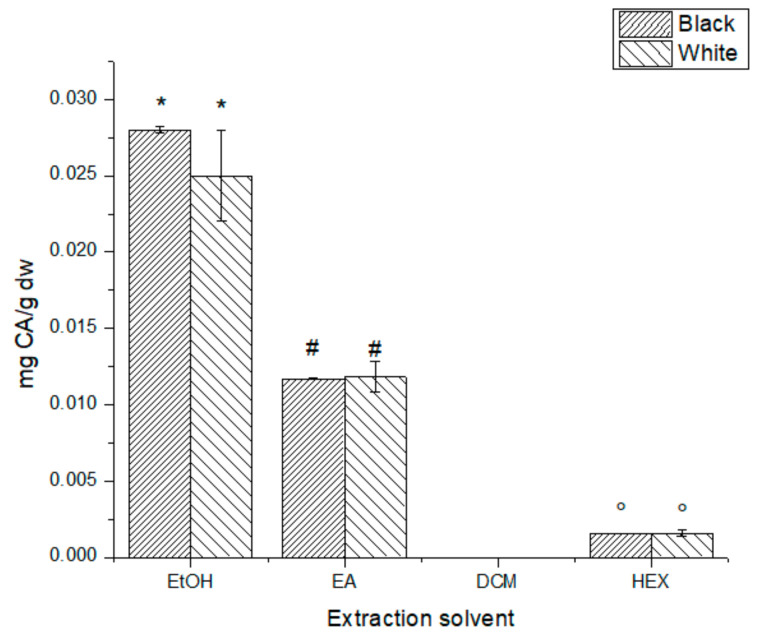
Caffeic acid content for all extracts tested. *, #, ° No significant difference (*p* > 0.05).

Regarding the caffeic acid content (Figure 6), despite the extract concentration injected (5000 mg L^−1^), the analytical signal was lower than the limit of detection in the calibration curve for the DCM extracts. Additionally, while there was no significant difference between extracts with the same type of solvent (B-EtOH and W-EtOH, for example), the difference between extracts done with different extraction solvents was statically significant (*p* < 0.05).

**Figure 7:**
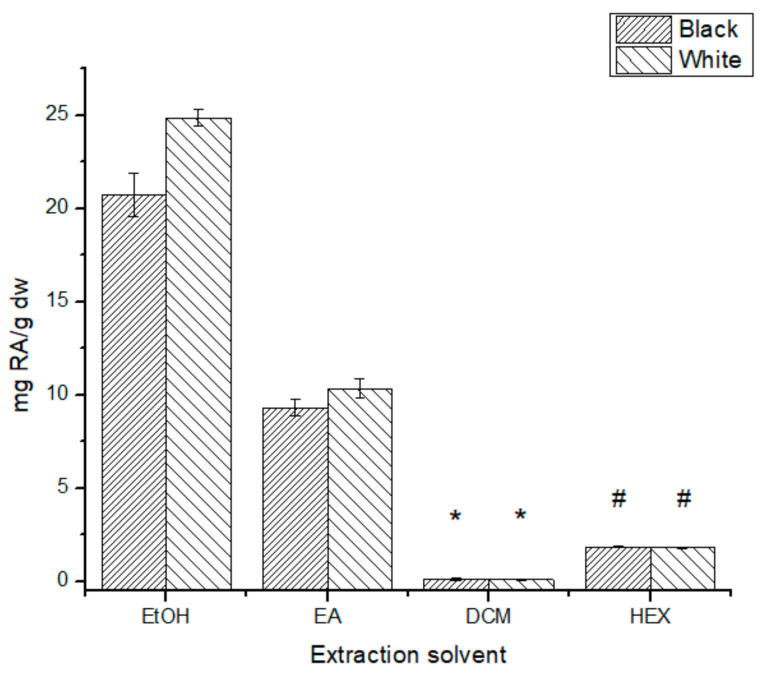
Rosmarinic Acid content for all extracts tested. *, # No significant difference (*p* > 0.05).

Rosmarinic acid, a caffeic acid derivative, showed a significantly higher concentration content in the ethanolic extracts (Figure 7), with the W-SPLE extract having the highest concentration at 24.9 ± 0.4 mg RA/g dw. The less polar extracts have no significant difference (*p* > 0.05) between the phenotypes, and all four of them have a concentration of RA under 2 mg RA/g dw.

### 3.4. Phenolic Identification

Additionally, in this work, 18 bioactive compounds were tentatively identified by UHPLC-HRMS (Q-Orbitrap) (Table 2).

The extracts performed with more polar solvents (EtOH and EA) had the highest number of identified compounds for both phenotypes. For instance, caffeic acid, rosmarinic acid, protocatechuic acid, *p*-coumaric acid, coumaric acid-*O*-hexose, kaempferol, and genistein were mainly found in these extracts. Salvianolic acid F isomer and dimethyl quercetin compounds were mostly found in the EA and DCM extracts. Finally, ferulic acid, luteolin-*O*-glucuronide, acetyl orientin, vitexin, and orientin were found in all the extracts under study. Instead, coumaroyl quinic acid was only found in the B-SPLE.

## 4. Discussion

### 4.1. Antioxidant Capacity

Comparing the results for each phenotype, ORAC index data (Figure 2) show a significant decrease in AC as the polarity of the solvent used decreases. Since the nature of the extracted compounds varies upon the polarity of the solvent, this effect could be bound to the solubility of the antioxidants extracted in ethanol and reaction medium. To amend this, a lipophilic ORAC assay should be used to corroborate this information [25]. Regardless of this, the ORAC-FL results for ethanolic extracts were ten-fold higher than those found for some teas and typical Chilean infusions (*Peumus boldus*: 2728 ± 122; *Haloplappus baylahuen*: 2250 ± 71), while the ORAC-PGR results were very similar with the teas tested [26]. Since ORAC-PGR is associated with the reactivity of the extract’s antioxidants, this result is not a very promising one, considering the good results on ORAC-FL; therefore, considering both ORAC methods might be a good indicator of the antioxidant quality of the extracts; thus, making the use of more than one procedure to estimate not only the total amount of antioxidants but also their reactivity essential. Although each method of AC quantification goes through a specific mechanism, a great fraction of the outcome seemed to be given by common denominators, most likely readily accessible hydroxyl groups of small molecules, of easy access, reacting with the free radicals present.

The ORAC-FL results obtained for the total extracts (done with AWA) are comparable to those found for some berries such as blueberries (26,642 ± 2257 µmol TE/100 g dw.) or maqui (19,850 ± 966 µmol TE/100 g dw) [27], both considered high antioxidant natural products.

Regarding the poor solubility of the antioxidants, it is important to say that, since the ORAC-PGR is an absorbance technique, its sensibility is lower than ORAC-FL, which could be the reason why no ORAC-PGR index was measured for the DCM and HEX extracts. Even though these difficulties arise, all the AC results point in the same direction: the ethanolic extracts of B-SPLE have the highest AC, which is comparable to that found for some berries [27].

Due to the different mechanisms involved in the ORAC and DPPH assays, it is possible to find different AC tendencies. This is the case for the DCM extracts, which have higher AC for the W-SPLE (13.94 ± 0.04%) than the B-SPLE (9.5 ± 0.7%) in DPPH data (Figure 3), contrary to the ORAC-FL data (Figure 1). At this point, it is remarkable that the B-EtOH extracts have the highest AC for all the AC assays, meaning that the antioxidants present in this extract are available to exert their antioxidant ability in both mechanisms.

Adımcılar et al. [28], performed a comparative study on 14 different *Salvia* species and reported a %Inhibition ranging from 3.76 ± 0.01 for *S. chionantha* to 93.2 ± 4.5 for *S aramiensis*. In comparison, the ethanolic extracts reported in the present study have an Inanition % similar to *S. hypargeia* (46.0 ± 1.4%). Kosar et al. [29] carried out a sequential Soxhlet extraction on *Salvia halophila* aerial parts, reporting a %Inhibition dependance on the extract type, similar to the dependance found in Figure 3, with the methanolic extract having a statically higher AC than the ethyl acetate extract.

Finally, the CAA experiments have a different tendency, with the W-SPLE showing higher AC than the B-SPLE. Wolfe et al. [21] reported a %CAA of 13.3 ± 1.1 for apple to be not statically different (*p* > 0.05) from that found for cranberry. This value is of the same order of the results for B-SPLE, but the results on W-SPLE are nearly twice those reported by Wolfe.

According to these AC analyzes, the chia leaf is a promising source of antioxidants, which has been discarded until now. Leaf AC expressed both quantity and reactivity qualities comparable to some fruits as berries or maqui, as well as some infusions such as tea, all of these being well known as good antioxidants.

### 4.2. Total Phenolic Content and Caffeic and Rosmarinic Acids Content

As said above, the FC assay could give a positive response to non-polyphenolic compounds capable of reducing the FC reagent [24] but, regardless of this, the data show a significantly higher TPC for B-EtOH extract (Figure 5), just as in all the AC assays. The solubility problem could be behind the low TPC found in the DCM and HEX extracts. Among the *Salvia* species studied by Adımcılar et al., the range of TPC was found between 6.47 ± 0.00 mg GAE/g dw and 260 ± 1 mg GAE/g dw, with *S aramiensis* at the top of the list. In this regard, even the hexane extracts (11.3 ± 0.4 g GAE/100 g dw B-SPLE; 11.8 ± 0.6 g GAE/ 100 g dw W-SPLE) are between the *Salvia* species with the highest amount of phenolics [28].

The global extraction experiment shows a mean of 2.0 ± 0.1 mg GAE/100 g dw for the B-SPLE and 1.71 ± 0.06 mg GAE/100 g dw for the W-SPLE, much lower than the 3116 ± 155 mg GAE/100 g dw reported for maqui [27], but it is noteworthy that the total polyphenols result of chia leaf and maqui obtained may differ in this way because fruits have a larger concentration of reducing sugars that may overexpress the obtained total phenolic values, unlike the chia leaf.

Caffeic acid is a phenolic acid synthetized from cinnamic acid that has previously been reported in chia leaves extracts [8]. This phenolic acid has some interesting properties in medicine [30,31,32], making chia leaves a potential source for future biological assays. Figure 6 shows similar results to the AC and TPC assays, but no significant difference was found between phenotypes upon a comparison with the same solvent. Again, the most polar solvents (EtOH and EA) show a higher content of CA due to the greater solubility of this compound and the greater extraction ratio. Ben Ferhat et al. reported CA content of *Salvia* species growing in different environments showing a CA variation upon the species and the location [33]. The CA concentration reported for this author (46.80 ± 0.33–695.04 ± 18.21 µg g^−1^ dw) is higher than the CA concentration found in *S. hispanica*, although Ben Ferhat et al. used a Soxhlet extraction procedure which varies significantly from the sequential extracts performed in the present work.

Rosmarinic acid is an ester of caffeic acid and 3,4-dihydroxyphenyllactic acid found in the Lamiaceae and Boraginaceae families, and similar to caffeic acid, this compound has been previously reported in chia leaves extracts [8]. Various biological properties have also been reported for this compound, anti-inflammatory and antioxidant among them [34]. Following the tendency, the ethanolic extracts had the highest content of RA (24.4 *±* 0.4 mg RA/g dw for W-SPLE and 21 *±* 1 mg RA/g dw for B-SPLE) of the samples analyzed in this work. The available literature on *Salvia* species shows a variation in RA and CA content upon the *Salvia* species and location [28,33]. Adımcılar et al. reported the RA content for several *Salvia* species [28], ranging between 1.08 ± 0.01 mg/g dw and 18.7 ± 2.0 mg/g dw, lower than the ethanolic extracts reported in this work. Ben Ferhat et al. [33] also reported RA content lower than the results on *S. hispanica*. Again, the extraction procedure used by Adımcılar et al. includes ultrasound assistance, which differs from the extraction procedure to achieve the sequential extracts. Kosar et al. [29] performed sequential extractions on *S. halophila* aerial parts, reporting a concentration of 48.90 ± 2.12 mg RA/g dw on the ethyl acetate extracts, followed by the methanolic (38.59 ± 0.22 mg/g dw) and the 50% methanolic (27.14 ± 0.89 mg/g dw), which are higher than the RA concentration on *S. hispanica*. This difference could be due to the different species and the extraction procedure.

### 4.3. Polyphenol Identification

As mentioned above, 18 phenolic compounds were tentatively identified in the sequential extracts performed. The results show that the B-SPLE had a higher number of compounds than the respective W-SPLE, with the ethanolic and DCM extracts topping the list.

Some of the identified compounds have been previously reported in chia leaf methanolic extracts [8], as well as in some other *Salvia* species [28,29,33]. As reported by Amato et al. [8], the majority were flavonoids and cinnamic acid derivatives. Most of the flavonoids were flavone derivatives, some of them acylated or glycosylated. Genistein and Naringenin were the only non-flavone ones. For cinnamic acid derivatives, almost all were RA or CA derivatives. Protocatechuic acid falls off these categories, being the only benzoic acid identified.

## 5. Conclusions

Nowadays, only chia seeds are commercialized since it is considered a natural source of omega-3 and omega-6, fiber, proteins, and natural antioxidants. Nevertheless, the chia plant may represent a by-product that could be considered a great source of bioactive compounds with unexplored potential medicine and food industry applications. In this work, bioactive compounds such as some cinnamic acids (caffeic acid, ferulic acid, or rosmarinic acid) and some flavonoids (vitexin or luteolin-*O*-glucuronide) were tentatively determined in all of the analyzed chia leaf extracts. The extracts performed with ethanol as the extraction solvent presented the highest chemical AC (ORAC and DPPH) and CAA, polyphenolic content, CA and RA content, and the number of phenolic and polyphenolic compounds tentatively identified. The CAA assay on the two extracts with the best results in the chemical analyzes, demonstrates that the global antioxidant capacity of chia leaf ethanolic extracts is promising for future in vivo assays.

## Figures and Tables

**Table 1:** Dry extract mass and extraction percentage for each extract obtained by successive maceration of chia leaves of black (B) and white (W) seed phenotype.

Extract	Extraction (%)	Mass (g)
B-EtOH	11.279	1.5507
B-AcET	2.389	0.3285
B-DCM	1.727	0.2374
B-HEX	35.790	4.9231
W-EtOH	14.668	0.9689
W-AcET	7.267	0.4800
W-DCM	3.043	0.2010
W-HEX	43.194	2.8532

**Table 2:** Tentative identification of bioactive compounds in chia leaf extracts of black (B) and white (W) seed phenotype.

Compound	Rt (min)	Formula	Delta *m*/*z*	B-EtOH	B-EA	B-DCM	B-Hex	W-EtOH	W-EA	W-DCM	W-Hex
Protocatechuic acid	4.44	C_7_H_6_O_4_	−0.78	+	+	+	+	+	−	n.d.	n.d.
Chlorogenic acid	5.78	C_16_H_18_O_9_	−0.20	−	n.d.	−	n.d.	n.d.	n.d.	n.d.	n.d.
*p*-coumaric acid	5.87	C_9_H_8_O_3_	−0.38	−	−	−	−	n.d.	n.d.	n.d.	n.d.
Coumaric acid-O-hexose	5.87	C_15_H_18_O_8_	−0.83	−	−	−	−	n2.	n.d.	n.d.	n.d.
Caffeic acid	6.48	C_9_H_8_O_4_	−0.63	++	+	+	+	−	−	−	−
Coumaroyl quinic acid	6.63	C_16_H_18_O_8_	−0.26	−	n.d.	+	n.d.	−	n.d.	+	n.d.
Orientin	7.21	C_21_H_20_O_11_	−0.79	++	+	+	+	+	−	+	−
Vitexin	7.64	C_21_H_20_O_10_	−0.95	++	++	++	++	+	+	+	+
Acetyl orientin	7.76	C_23_H_22_O_12_	−0.87	+	−	−	−	−	n.d.	−	−
Luteolin-O-glucuronide	7.94	C_21_H_18_O_12_	−0.62	+	+	+	+	+	−	+	+
Sinapic acid	9.00	C_11_H_12_O_5_	−0.47	n.d.	n.d.	−	−	−	−	n.d.	n.d.
Rosmarinic acid	9.03	C_18_H_16_O_8_	−068	++	++	++	++	+	+	−	+
Ferulic acid	9.50	C_10_H_10_O_4_	−1.19	+	+	+	+	−	−	+	+
Kaempferol	10.45	C_15_H_10_O_6_	−0.51	−	−	+	+	n.d.	n.d.	−	−
Genistein	11.56	C_15_H_10_O_5_	−0.75	−	−	+	+	n.d.	n.d.	−	−
Naringenin	11.66	C_15_H_12_O_5_	−0.50	−	−	++	++	+	+	+	+
Salvianolic acid F isomer	11.84	C_17_H_14_O_6_	−0.14	−	n.d.	−	−	+	+	−	−
Dimethyl quercetin (quercetin dimethyl ether)	11.86	C_17_H_14_O_7_	−0.37	+	+	++	++	++	++	+	+

++ area > 10^+7^, + area > 10^+6^, − area > 10^+5^, n.d. not detected.

## Data Availability

Data is contained within the article and supplementary material.

## Supplementary Materials

The following are available online at https://www.mdpi.com/article/10.3390/antiox10071151/s1, Table S1: Phenolic accurate mass database employed for chia methanolic extract screening.
