# Supplementary material for: Determination of Bioactive Compounds in Sequential Extracts of Chia Leaf (Salvia hispanica L.) Using UHPLC-HRMS (Q-Orbitrap) and a Global Evaluation of Antioxidant In Vitro Capacity
Source: Antioxidants (Basel). 2021 Jul 20;10(7):1151. doi: 10.3390/antiox10071151 (PMC8301112; doi:10.3390/antiox10071151)
Supplement: Supplementary file 1 [file antioxidants-10-01151-s001.zip › antioxidants-1278958-supplementary.pdf]

Supplementary Materials

Table S1: Phenolic accurate mass database employed for chia leaf methanolic extract screening.

Compound	Formula	[M-H]$^-$ exact *m*/*z* value
2',3,4,4'-Tetrahydroxychalcone	C15H12O5	271.0612
4-coumaroyl-CoA	C30H42N7O18P3S	912.1447
7-Hydroxyisoflavone	C15H10O3	237.0557
Acetyl orientin	C23H22O12	489.1039
Apiforol	C15H14O5	273.0769
Caffeic acid	C9H8O4	179.0350
Catechin	C15H14O6	289.0718
Chlorogenic acid	C16H18O9	353.0878
Cinnamic Acid	C9H8O2	147.0452
Citric acid	C6H8O7	191.0197
Coumaric acid-O-hexose	C15H18O8	325.0929
Coumaroyl quinic acid	C16H18O8	337.0929
Daidzein	C15H10O4	253.0506
Dihydrokaempferol	C15H12O6	287.0561
Dihydromyricetin	C15H12O8	319.0459
Dihydroquercetin	C15H12O7	303.0510
Dimethyl quercetin	C17H14O7	329.0667
Ellagic Acid	C14H6O8	300.9990
Ellagic acid glucoside	C20H18O13	465.0675
Eriodictyol	C15H12O6	287.0561
Eucine/Esoleucine	C6H13NO2	130.0874
Ferulic acid	C10H10O4	193.0506
Genistein	C15H10O5	269.0456
Hesperidin	C28H34O15	609.1825
Hesperitin	C16H14O6	301.0718
Kaempferol	C15H10O6	285.0405
Leucoanthocyanidin	C15H14O8	321.0616
Liquiritigenin	C15H12O4	255.0663
Luteoforol	C15H14O6	289.0718
Luteolin	C15H10O6	285.0405
Luteolin-7-O-glucoside	C21H20O11	447.0933
Luteolin-O-glucuronide	C21H18O12	461.0726
Malonyl-CoA	C24H38N7O19P3S	852.1083
Medicarpin	C16H14O4	269.0819

Myricetin	$C_{15}H_{10}O_8$	317.0303
Naringenin	$C_{15}H_{12}O_5$	271.0612
Orientin	$C_{21}H_{20}O_{11}$	447.0933
p-Coumaric acid	$C_9H_8O_3$	163.0401
Phenylalanine	$C_9H_{11}NO_2$	164.0717
Polydatin	$C_{20}H_{22}O_8$	389.1242
Protocatechuic acid	$C_7H_6O_4$	153.0193
Quercetin	$C_{15}H_{10}O_7$	301.0354
Resveratrol	$C_{14}H_{12}O_3$	227.0714
Rosmarinic acid	$C_{18}H_{16}O_8$	359.0772
Salvianolic acid F isomer	$C_{17}H_{14}O_6$	313.0718
Sinapic acid	$C_{11}H_{12}O_5$	223.0612
Trihydroxychalone	$C_{15}H_{12}O_4$	255.0663
Tryptophan	$C_{11}H_{12}N_2O_2$	203.0826
Vitexin	$C_{21}H_{20}O_{10}$	431.0984
